# Comparison between the surgical scalpel and carbon dioxide laser in managing excessive gingival display using lip repositioning technique: a randomized controlled clinical study

**DOI:** 10.1186/s40902-025-00475-8

**Published:** 2025-08-14

**Authors:** Sara Alkari, Omar Hamadah, Steven Parker

**Affiliations:** 1 Faculty of Dentistry, Damascus University, Damascus, Syrian Arab Republic; 2 De Montfort University, Leicester, UK

**Keywords:** Carbon dioxide laser, Excessive gingival display, Lip repositioning, Vestibuloplasty

## Abstract

**Background:**

Excessive gingival display is a common and often distressing aesthetic concern among patients. With the increasing emphasis on dental aesthetics, it has become essential to investigate and develop various treatment modalities to address patients’ expectations. Achieving a balanced and attractive smile has therefore become a key challenge for dental practitioners *worldwide*.

**Methods:**

This study is a single-center, parallel, randomized controlled open-label trial aimed to compare performing lip repositioning surgery (LRS) utilizing carbon dioxide (CO₂) laser with conventional scalpel in managing excessive gingival display (EGD) (4–6 mm) with special consideration to lip dimensions, and 20 participants were included in this study setting in the Faculty of Dentistry, 70% females and 30% males, with an average age of 26.4 and 24.8 years in laser and scalpel groups, respectively, randomly allocated into two groups. All patients fulfilled the study.

A partial thickness flap was excised from the vestibule, with both edges of the donor wound closed using sutures; clinical measurements were recorded preoperatively and followed up for 6 months.

**Results:**

Postoperative pain was significantly reduced in the laser group; both groups demonstrated a significant decrease in gingival display at 6-month post-operation: external upper lip length was significantly longer in the laser group in smile position at 1-month post-operation, and internal upper lip length at rest was significantly longer in the scalpel group at 3-month post-surgery (significance level was set at *P* < 0.05).

**Conclusions:**

The CO₂ laser is an effective, safe, and conservative alternative to the scalpel in performing LRS, offering decreased pain and improved visualization. Adequate training in laser techniques and safety is essential.

**Trial registration:**

The study is registered at International Standard Randomized Controlled Trial, registration number ISRCTN.

## Introduction

A smile is one of the most significant facial expressions in human social interaction. However, the concept of an attractive smile remains complex. An aesthetically pleasing smile is typically achieved when the teeth, gingiva, and lips are in harmony. Among the common esthetic concerns that compromise smile attractiveness is excessive gingival display (EGD), widely known as a “gummy smile,” which presents a clinical challenge for dentists [1]. Therefore, thorough understanding and accurate diagnosis are essential in each case to ensure predictable treatment outcomes. Moreover, a high smile line is a frequent aesthetic complaint, as it negatively impacts patients’ self-esteem in both social and professional contexts. The exposure of more than a 2-mm band of gingiva during smiling is regarded as indicative of EGD [2]. Across individuals, the prevalence is 10.5% to 29% with an evident gender difference with a 2:1 female tendency [3]. EGD has a multifactorial etiology; hence, accurate identification of the underlying cause is crucial for a reliable diagnosis. Contributing factors may include excessive vertical maxillary growth, short or hypermobile upper lips, short clinical crowns, gingival hyperplasia, or a combination of these elements. A precise diagnosis enables meticulous planning and realistic outcome prediction, in alignment with the patient’s expectations [4, 5].

The external upper lip length, measured from the base of the nose to the inferior border of the upper lip, plays an essential role in smile esthetics. A normal range in young adults is between 20 and 24 mm and is considered normal in young adults. An upper lip measuring less than 20 mm is considered short and may contribute to EGD [6]. EGD can be classified into three degrees of severity (mild: 2–4 mm, moderate: 5–8 mm, and severe: more than 8 mm), based on the amount of exposed gingiva during a maximal smile. This classification helps clinicians in treatment planning and guiding appropriate interventions [7]. Furthermore, classifying the degree of EGD should also be taken into account to match the treatment plan to optimal and effective outcomes.

Lip repositioning surgery (LRS), first described by Rubinstein and Kostianovsky in the 1970 s, is a minimally invasive treatment for EGD. It is widely considered an effective, safe, and predictable treatment approach, with an average of 2.71 mm in decreasing the amount of exposed gingiva. The fundamental principle of LRS is to limit upper lip elevation by reducing muscle activity, which is achieved by removing a strip of mucosa from the buccal vestibule. Over time, several modifications to the original technique have been proposed, including myotomies and subperiosteal dissection of the gingiva [8, 9].

Dental lasers may offer significant benefits, such as high levels of precise hemostasis, a sterile surgical field, minimal postoperative pain, and uneventful wound healing when used in the delivery of oral soft-tissue surgery. Given the high-water content of oral soft tissues, the CO₂ laser, with its 10,600-nm emission wavelength, has a strong absorption peak, making it particularly suitable for such procedures [10, 11]. However, laser-assisted lip repositioning studies remain limited and are often with small sample sizes; more research is needed to provide evidence on using lasers in these contexts.

In the light of the facts mentioned earlier, this study was conducted to evaluate the effectiveness of the CO_2_ laser in lip repositioning surgery to assess its potential advantages in managing EGD of soft-tissue factorial nature, in comparison with the conventional surgical scalpel, guided by evidence-based recommendations from the existing literature.

## Materials and methods

This study was designed as a single-center parallel randomized controlled open-label trial. The ethical approval for the study protocol was obtained from the Ethics Committee of Damascus University (date: 9/5/2022/no: 2609). The study was registered at the International Standard Randomized Controlled Trial, registration number: ISRCTN11661014.

(https://www.isrctn.com/ISRCTN11661014). Informed consent for participating in the study was obtained from every patient.

Participants were randomly allocated into two groups using a simple randomization method, where each participant drew sealed, opaque envelopes from a box (n: 10; n: 10); each envelope contains a numbered paper (1 or 2). Twenty patients were randomized; the same patients were analyzed. No changes were made to methods after trial commencement.

### Consent for publication

A prospective study was undertaken; the sample size calculation was performed using G*Power version 3.1.9.4, to detect different amounts of gingival display between groups [12], assuming a large effect size (Cohen’s d = 0.8), a significance level (α) of 0.05, and a desired power of ≥ 0.80. Based on these parameters, the minimum required sample size was determined to be 16 participants (8 per group). To enhance the statistical robustness of the study and account for potential dropouts or variability, the sample size was increased to 20 participants (10 per group). The predicted sample size was 16, 8 patients in each group. Based on these results, we have selected a sample size of 20, 10 in each group; all of them were presented to our department at the university or referred from other departments between 2022 and 2024, with a chief complaint of an unpleasing smile, to 2 times more than the natural distance, which is 6–8 mm.

Following a discussion of the procedure, its pros and cons, and potential postsurgical expectations, patients received an initial consultation on eligibility and ensured that their condition met the inclusion criteria. This involved analyzing smile dimensions to exclude dentoalveolar origin, measuring clinical crown lengths of the teeth to rule out short clinical crowns as a factor. This was done by placing a periodontal probe at the midpoint of the buccal surface of each tooth, parallel to its longitudinal axis, and recording the distance in millimeters between the gingival margin and the incisal edge. Dividing the face into three equal parts checks for imbalance. Standardized photographs and cephalometric images were also taken to rule out skeletal causes and to ensure the EGD case was of soft-tissue nature. Key anatomical landmarks, such as maxillary height and the SNA angle, were analyzed. The SNA angle, which normally measures around 82°, is formed by 3 points in which (S) is the center of the sella turcica, (N) is the most anterior point of the nasofrontal suture, and (A) is the most posterior point on the anterior maxillary surface. Upper lip hypermobility was assessed by measuring lip movements from the rest to the maximal smile positions. Lip elevator muscles were considered hyperactive when their elevation was between 6 and 8 mm from the rest position. All patients chose lip repositioning surgery.

### Participant selection

#### Inclusion criteria

1. Adult patients aged between 18 and 38 years
2. Good periodontal health based on clinical examination (no periodontal pockets, no bleeding, healthy gingival appearance)
3. Systemically healthy individuals, classified as ASA physical status I or II (i.e., patients without systemic disease or with mild, well-controlled systemic conditions)
4. Excessive gingival display ranging between 4 and 6 mm caused by a short or hyperactive upper lip.

#### Exclusion criteria

1. Smokers.
2. Pregnant or lactating women.
3. Vertical maxillary excess of more than 6 mm.
4. Systemic disease or cases that forbid local anesthesia.

The sample of 20 patients was randomly assigned to one of two groups; each patient was asked to choose a piece of paper numbered 1 or 2, for which the first one referred to Group no. 1: 10 patients with EGD, treated with lip repositioning surgery by removing a partial thickness flap utilizing the conventional scalpel. Number 2 referred to Group no. 2: 10 patients with EGD, treated with lip repositioning surgery by removing a partial thickness flap utilizing the CO_2_ laser (CO_2_ laser; E301, Beijing, China, wavelength 10.600 nm).

**Fig. 1:**
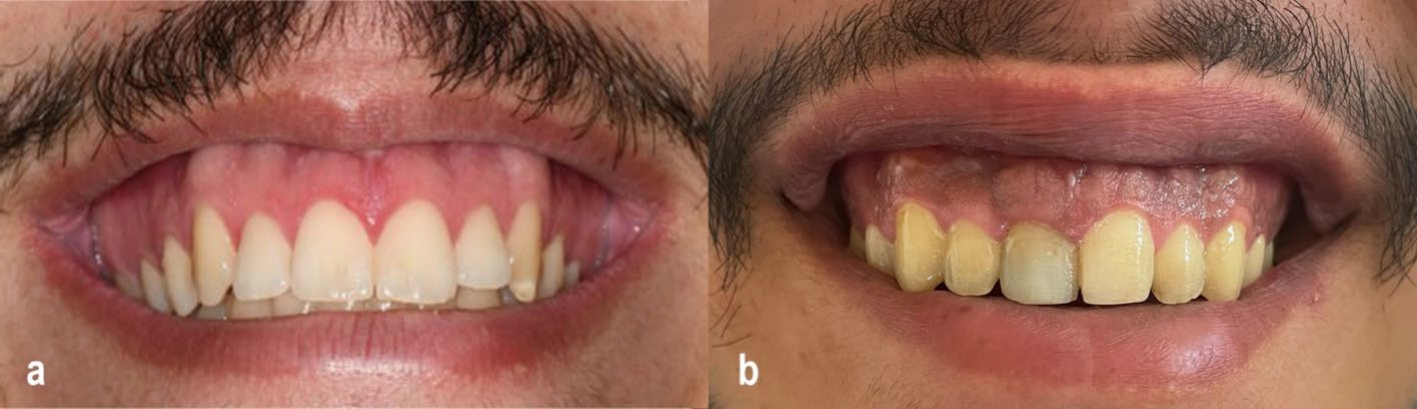
**a** Preoperative photograph of a patient from the CO₂ laser group demonstrating excessive gingival display (4–6 mm) during maximal smile prior to lip repositioning surgery. **b** Preoperative photograph of a patient from the conventional scalpel group showing excessive gingival display (4–6 mm) during maximal smile before undergoing lip repositioning surgery

An informed consent was obtained from each patient, irrespective of which group. Prior to surgery, intra- and extraoral photographs were taken using a 7500D Nikon camera (Nikon, Tokyo, Japan), and all measurements were recorded (Fig. 1a, b).

**Fig. 2:**
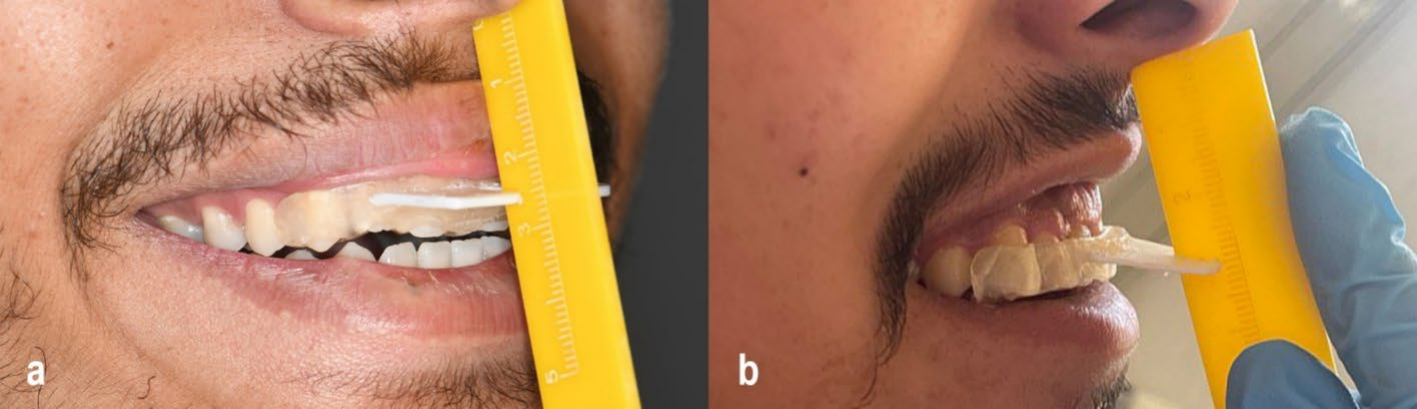
**a** Measurement of external upper lip length during smile using a custom-designed ruler to ensure consistent positioning in a CO₂ laser group patient. **b** Measurement of external upper lip length during smile using the same custom-designed ruler in a conventional scalpel group patient

The amount of exposed gingiva was recorded using a gingival probe. Following several previous studies, measurements were taken from the inferior border of the upper lip to the zenith point on the gingival margin, starting at the central incisors and ending at the second premolars on each side. Assessments were performed before surgery and at 1, 3, and 6 months postoperatively [13]. A custom-made appliance with a hollow midpoint and horizontal extraoral arm was fabricated after taking an impression of the maxillary teeth for each patient. A sterile millimetric graduated ruler was inserted vertically into the hollow part of the appliance to ensure consistent measurements; this technique was developed to guarantee measuring the upper lip length from the same position before surgery. In all the recall appointments, patients were instructed to bite in normal occlusion. The external upper lip length was recorded both at rest and maximal smile, preoperatively, and at 1-, 3-, and 6-month post-operation (Fig. 2a, b).

The internal upper lip length (vestibular depth) was also recorded clinically using a specially designed graduated ruler with a hollow midpoint, which allowed exclusion of the upper labial frenum and ensured consistent positioning for measuring [14].

### Intervention

#### Operative procedure

Prior to surgery, both external and internal oral tissues were disinfected with 2.0% chlorhexidine solution and 0.12% chlorhexidine rinse for 1 min. Local anesthesia was administered by infraorbital infiltration injection in both sides using 2% lidocaine with 1:80,000 epinephrine.

**Fig. 3:**
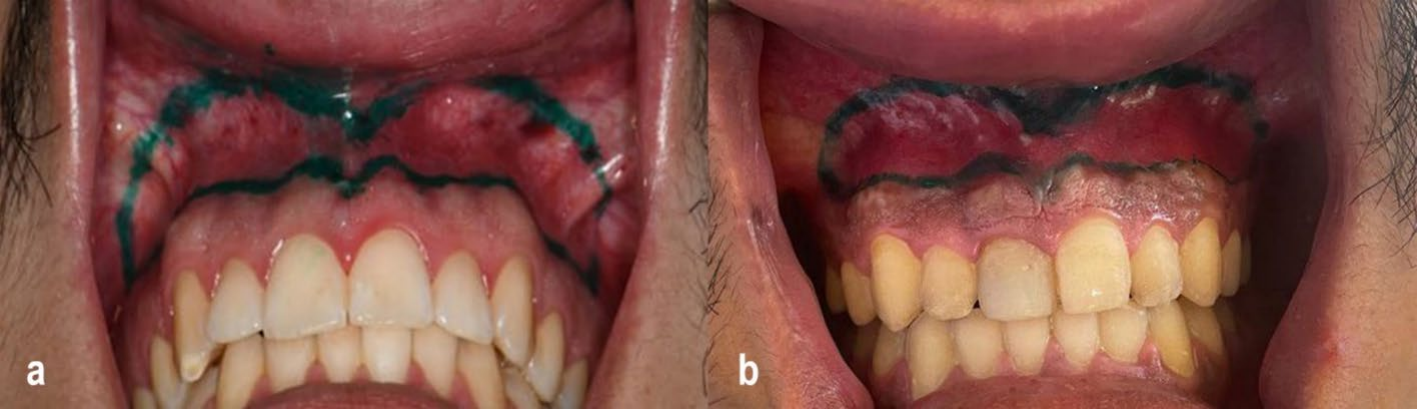
**a** Intraoperative marking of the surgical flap in a CO₂ laser group patient. The inferior incision is made 1 mm apical to the mucogingival junction; the superior incision is 10–12 mm above and parallel to the first. **b** Intraoperative marking of the surgical flap in a conventional scalpel group patient. The inferior incision is made 1 mm apical to the mucogingival junction; the superior incision is 10–12 mm above and parallel to the first

The surgical procedure involved marking the incision lines on the dry mucosa in each patient using a sterile surgical marking pen (Fig. 3a, b).

A horizontal partial-thickness incision, extending from the mesial line of the right maxillary second premolar to the left maxillary second premolar, was made 1 mm coronal to the mucogingival junction (MGJ); a V-shaped incision was made in the upper lip frenum area to facilitate guaranteeing labial midline position with punctual lip symmetry and accurate closure.

**Fig. 4:**
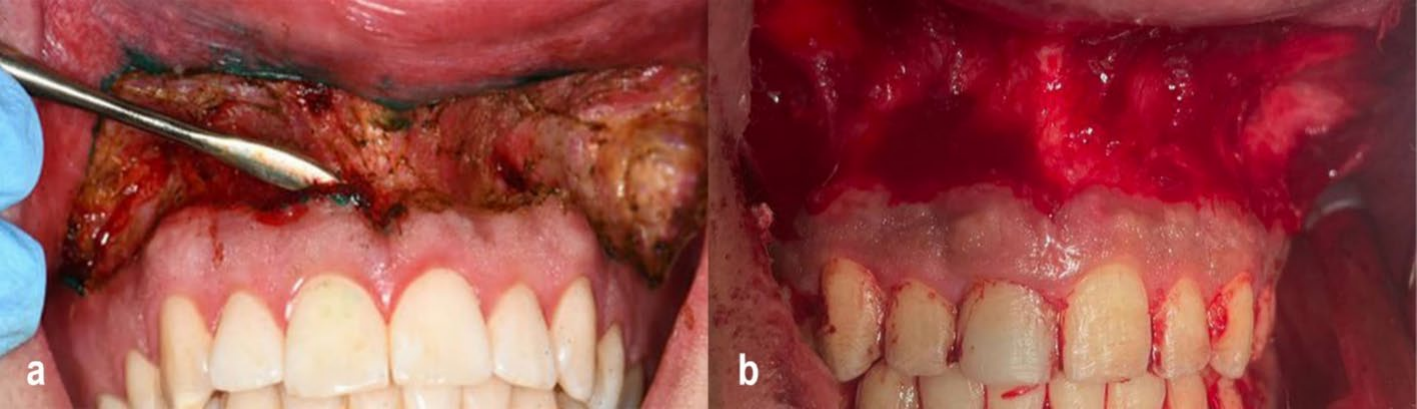
**a** Post-flap removal view in a CO₂ laser group case showing a clean surgical field with minimal bleeding due to laser-induced hemostasis. **b** Post-flap removal view in a conventional scalpel group case demonstrates noticeable bleeding due to the high vascularity of the upper lip region

**Fig. 5:**
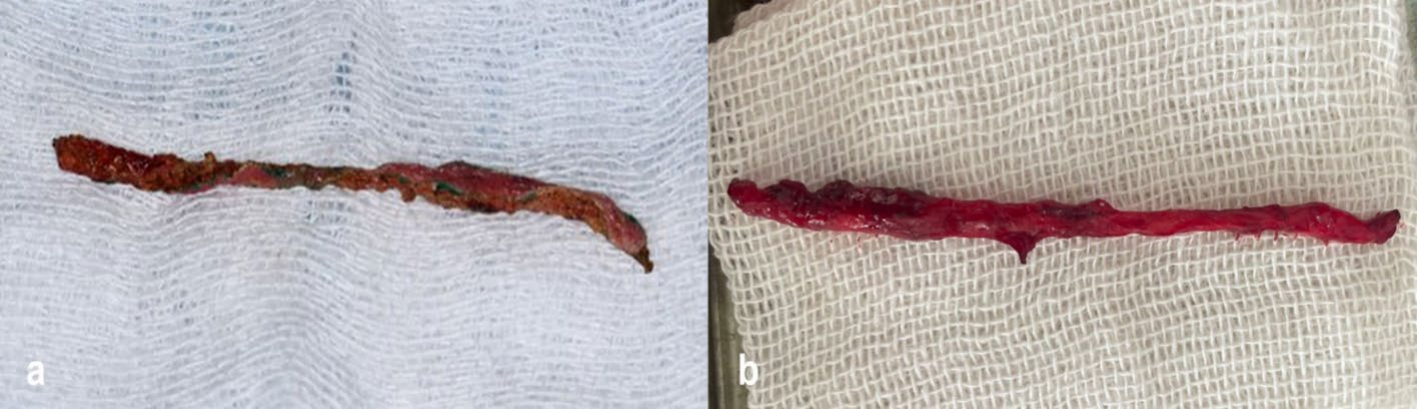
**a** A strip of mucosa removed using a CO₂ laser as part of the partial-thickness flap technique. **b** A strip of mucosa removed using a conventional scalpel in the corresponding group

A second horizontal incision was then made in the labial mucosa, positioned approximately 10 to 12 mm apical to the first incision. Finally, the two incisions were connected at the mesial line angles of the right maxillary first molar in an oval shape (Fig. 4a, b), and.

then a strip of mucosa was fully removed (Fig. 5a, b).

In Group 1, all previous surgical steps were performed using a Bard-Parker no. 15 stainless steel scalpel blade.

The same surgical technique was performed in Group no. 2 utilizing the CO_2_ laser. All the clinical staff were aware of laser safety measures (controlled area, wavelength-specific eyewear) and the wearing of high-filtration face masks to address the laser surgical plume. A laser test fire was performed to determine beam patency.

Laser operating parameters were as follows:

**Table:** 

Wavelength	10,600 nm
Application	Non-contact
Emission mode	Super pulsed
Pulse width	300 µs
Pulse interval	500 µs
Frequency	1250 Hz
Beam spot size	0.5 mm (sq. cm)
Average power	2.2 W
Peak power	6 W

#### CO₂ laser emission mode

In some laser systems, the super pulsed mode refers to the emission of very short, high-frequency pulses generated by modulating the electrical pumping mechanism. Unlike gated or chopped modes, often inaccurately referred to as “pulsed” true super pulsed lasers can achieve high peak power and power density while maintaining low average power. This enables deeper energy penetration into tissues with minimal thermal buildup, making super pulsed lasers particularly suitable for precise and controlled surgical applications. Some devices also allow the emission of grouped pulses, known as pulse trains, further enhancing energy delivery efficiency [11]. The surgical procedure involved applying the laser beam at the outlined borders of the proposed flap in order to reduce the tension and make the mucosal strip dissection smoother; the laser beam was then advanced along the oval-shaped line from the first side, adopting a 45° angled handpiece and with normal hand speed in order not to cut the strip or deepen the flap.

Laser-tissue distance was maintained by the laser hand-piece spacer. Once the tissue strip had been removed, a no. 15 scalpel blade was used in gentle peeling movements; hence, a slight amount of bleeding was induced at this stage to accelerate the healing process; hitherto, surgical field visualization and hemostasis through laser use had been maintained.

**Fig. 6:**
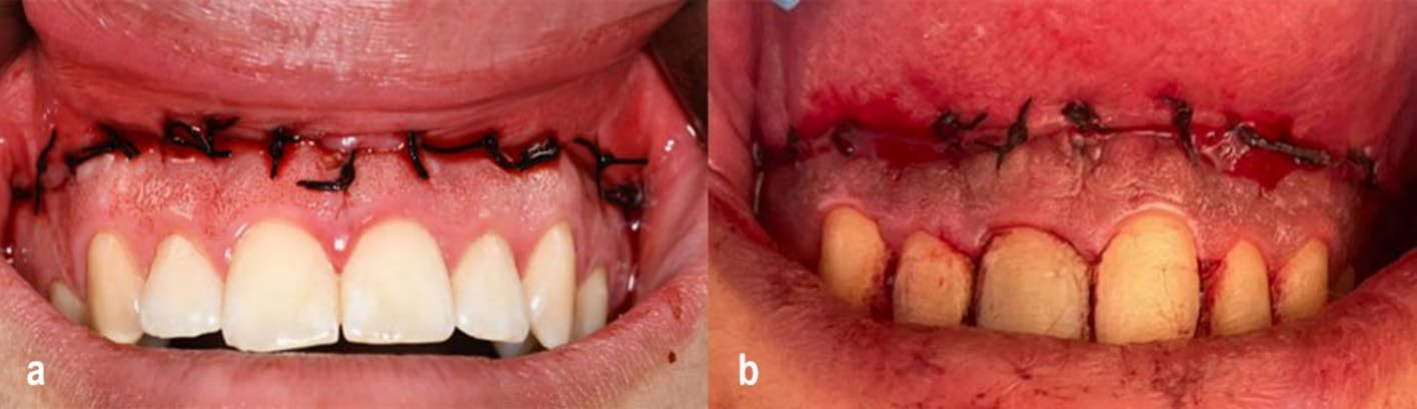
**a** Suturing of wound margins in a CO₂ laser group patient using 4–0 silk sutures in an interrupted technique. **b** Suturing of wound margins in a conventional scalpel group patient using the same interrupted technique with 4–0 silk sutures

In both groups, after the strip had been removed, a durable suturing was performed starting with a guiding suture in the midline area, indicated by a V-shaped index in the middle of the flap. Two further sutures were placed in the canine area on each side to assist in achieving reliable lip symmetry and ensure proper alignment of the lip midline with the teeth. Several interrupted 0000 silk sutures were used to close the rest of the wound area and to stabilize the upper lip in its new position (Fig. 6a, b).

A postoperative written prescription was given to each patient:

(i) Nonsteroidal anti-inflammatory drugs (ibuprofen 400 mg was administered four times daily for 2 days).
(ii) A total of 0.12% chlorhexidine mouthrinse (rinsing two times for 30 s per day, for 1 week).

### Postoperative instructions

A) Patients were instructed to apply ice packs over the upper lip intermittently for several hours post-surgery.
B) Stick to soft food for the first week and avoid any activities that could cause mechanical trauma to the surgical area.
C) Prevent wide smiling and minimize lip movement when talking for the first 2 weeks after surgery.
D) Avoid brushing around the wound for 2 weeks.
E) Take prescribed analgesics to manage any postoperative pain and, if ineffective, ask your dentist.
F) Sutures to be removed after 14 days.

### Postoperative assessment

#### Follow-up

The intensity of postoperative pain was recorded 24 h after surgery by asking patients to choose a number from 0 to 10 on a Numeric Pain Intensity Scale which is a type of VAS (visual analog scale), where 0 refers to no pain, 1–3 mild pain, 4–6 moderate pain, and 7–10 severe pain [15]. Pain level was recorded at a single time point based on previous studies indicating that VAS is not linear and does not accurately track changes in pain intensity over time [16].

Postsurgical edema usually peaks on the third day post-operation before starting to subside by day 4 [17–20]. The occurrence of edema was assessed at its peak on the third day postoperatively and on day 7 by recording “Yes” if edema was present or “No” if absent. Edema was observed in all patients of both groups on day 3 and was absent on day 7, with visually noticeable differences between the groups. Since this is the first randomized controlled clinical trial comparing CO₂ laser and scalpel techniques in this surgery, we recommend future researchers to study these differences quantitatively or volumetrically for a more precise understanding.

The amount of exposed gingiva, the upper lip’s external and internal length, was recorded according to the same methods used in the diagnosis appointment.

**Fig. 7:**
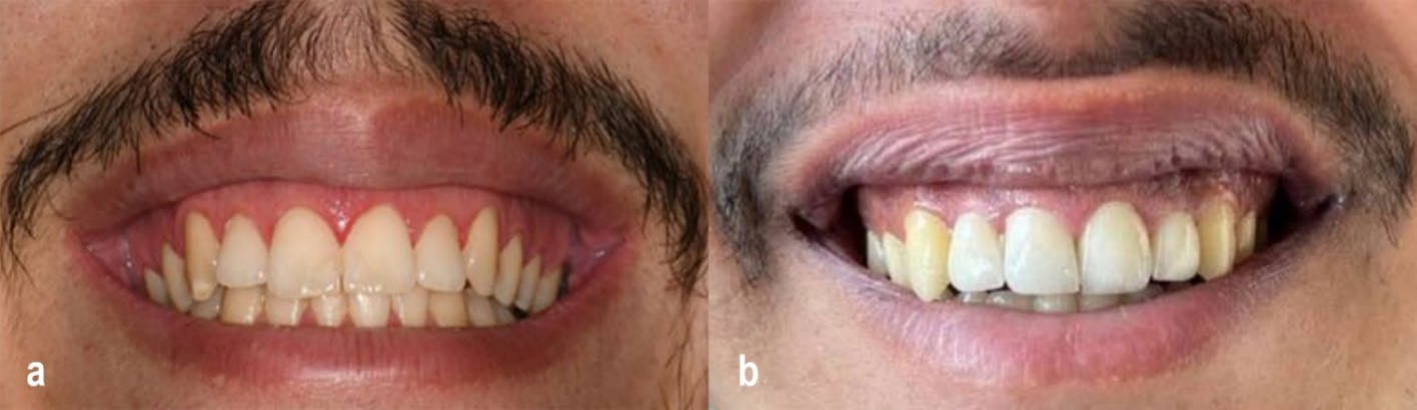
**a** Six-month follow-up in a CO₂ laser group patient showing an esthetically improved gingival display during maximal smile. **b** Six-month follow-up in a conventional scalpel group patient demonstrating significant improvement in gingival display during maximal smile

Patients were followed up within 1-, 3-, and 6-month post-surgery (Fig. 7a, b), all the measurements were recorded by the same examiner (the researcher), and clinical photographs were taken at all follow-up appointments.

#### Statistical analysis

The Statistical Package for the Social Sciences (SPSS) was used, version 25, to analyze the data as follows:

- Descriptive statistics: By displaying the mean and standard deviation of the variables according to the two study groups.
- Normal distribution test: Using the Shapiro–Wilk test when the sample size is smaller than 50 in order to find out the most appropriate tests for the study
- Difference tests: The independent *T*-test and its nonparametric alternative, the Mann–Whitney test, to study the differences between the two groups in the study variables; the paired samples *T*-test and its nonparametric alternative, the Wilcoxon signed-ranks test, for comparisons over time within the same group; and the analysis of variance for repeated measures and its nonparametric alternative, the Friedman test, to confirm the presence of significant differences in the studied group over time.

The testing was performed at pre-set alpha of 0.05, meaning that a significant difference exists with 95% confidence.

## Results

**Fig. 8:**
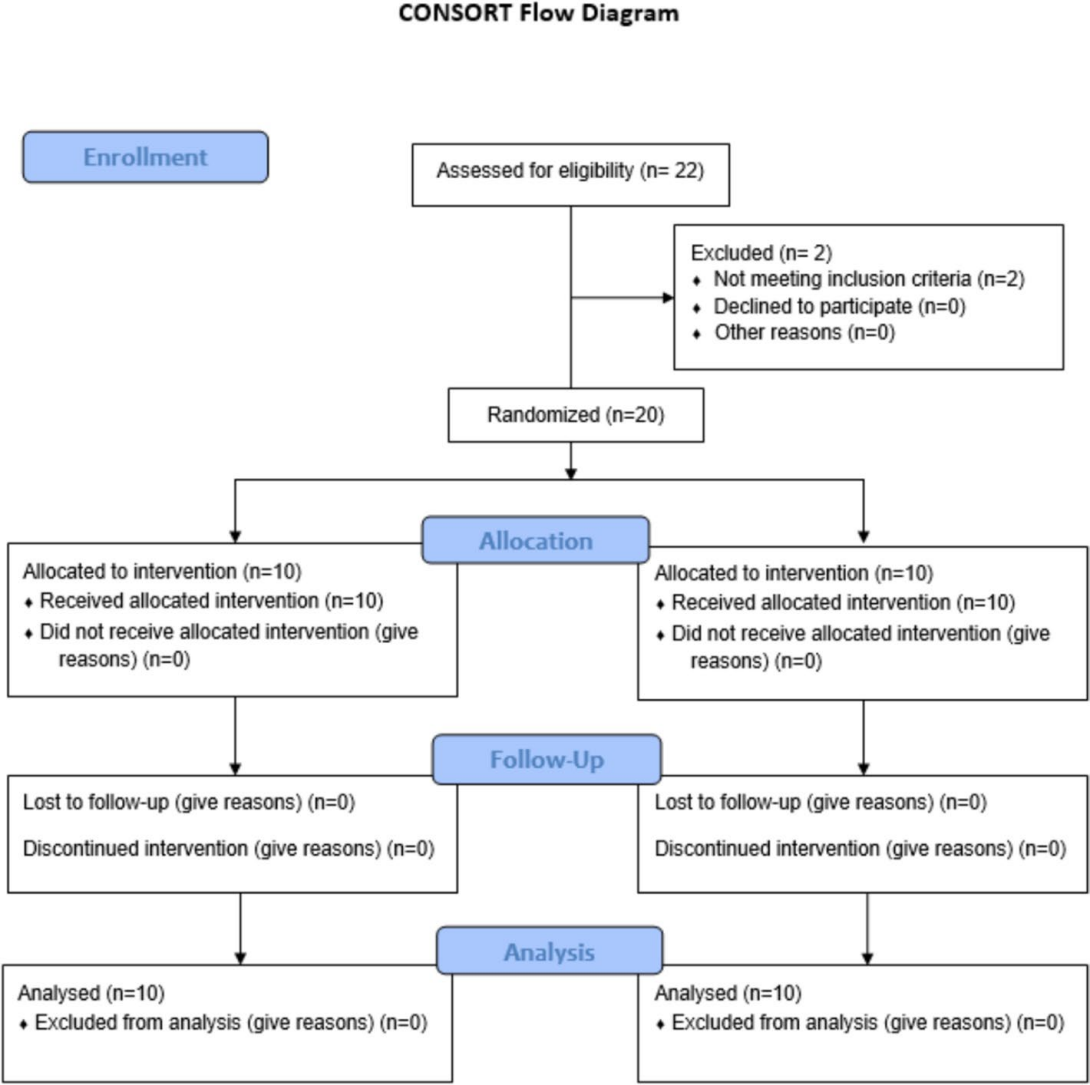
CONSORT flow diagram of participant recruitment and allocation. A total of 22 patients were assessed for eligibility; 2 were excluded due to skeletal causes of excessive gingival display. Twenty participants were randomized equally into two groups: Group 1 underwent lip repositioning surgery using a conventional surgical scalpel, while Group 2 was treated with carbon dioxide (CO₂) laser. All participants received the allocated intervention and completed the study without loss to follow-up or exclusion from analysis

Twenty patients, 15 females and 5 males, aged between 18 and 30 years, with EGD of 4 to 6 mm caused by soft tissue factors were included in this study and followed up within 1, 3, and 6 months postoperatively (Fig. 8).

The results demonstrated that the carbon dioxide laser group gender tendency was 70% females to 30% males, while in the conventional surgical scalpel group, the ratio was 80% females to 20% males. The age average was 26.4 years in the laser group compared to 24.8 years in the conventional scalpel group, with no significant differences in gender or age between the two groups (Table 1).

**Table 1:** Description of the study sample by gender and age

		Laser group		Scalpel group		*p*-value
		*n*	%	*N*	%	
Gender	Male	3	30%	2	20%	0.606a
	Female	7	70%	8	80%	
Age	Mean ± SD	26.40 ± 2.67		24.80 ± 4.66		0.362b

^a^Chi-square test. ^b^Independent samples *T*-test

Patients in both groups were satisfied with the overall procedure, but most of them complained of pain and edema in the initial phase post-surgery; 70% of laser group patients reported mild pain and 30% stated no pain in the day next to the surgery with mean value 0.70, while the percentage in scalpel group was 70% for mild pain, 20% for moderate, and only one patient suffered from severe pain with mean value 1.40. The 95% confidence interval for the mean difference (− 1.26 to − 0.14) does not contain zero; this means that the difference is not only statistically significant but also potentially clinically relevant. The lower pain levels in the laser group may therefore represent a meaningful clinical advantage in terms of patient comfort during the early postoperative phase. However, the CO_2_ laser group demonstrated significant statistical differences in reducing post-operation pain, in comparison with the scalpel group (*p*-value < 0.05) (Table 2.).

**Table 2.** Description of pain level and significant differences between the two groups

	Laser group			Scalpel group			Mean difference	95% confidence interval of the difference		*p*-value^a^
	Mean	*SD*	Median	Mean	*SD*	Median		Lower	Upper	
Pain level 24 h after surgery	0.70	0.48	1	1.40	0.70	1	− 0.70	− 1.26	− 0.14	0.017*

^a^Mann-Whitney test. Significant in *, 0.05

The results also elaborated a significant reduction in the amount of gingival display in each group over the evaluation periods; the average reduction in exposed gingiva was 1.96 mm in the laser group and 1.44 mm in the surgical scalpel group 6-month post-operation, with no significant differences between the two groups. The results did not show statistically significant differences between the laser and scalpel groups in terms of gingival exposure at any of the evaluated time points. Moreover, the 95% confidence intervals for all mean differences included zero, indicating that the differences were not clinically significant either. This supports the conclusion that the observed differences between the two groups are not clinically meaningful (Table 3).

**Table 3:** Descriptive and inferential analysis of gingival exposure between CO₂ laser and scalpel groups across evaluation periods

Evaluation period	Laser mean	Laser SD	Laser median	Scalpel mean	Scalpel SD	Scalpel median	Mean difference	95% confidence interval of the difference lower	95% confidence interval of the difference upper	*p*-value
Before surgery	4.80	0.55	4.77	4.61	0.67	4.64	0.19	− 0.39	0.77	0.497a
1 month	1.13	1.38	0.32	1.25	1.21	1.41	− 0.13	− 1.35	1.09	0.784b
3 months	2.18	0.92	1.86	2.55	1.06	2.09	− 0.37	− 1.31	0.56	0.413a
6 months	2.84	0.86	2.95	3.17	1.10	2.91	− 0.34	− 1.27	0.59	0.457a

^a^Independent samples *T*-test. ^b^Mann-Whitney test

Analysis of the external upper lip length data revealed that the laser group had a significantly longer external upper lip length, when smiling only 1 month after the procedure, where it was 2.30 mm greater than the scalpel group (*p*-value < 0.05). At other evaluation periods, the differences were as follows: 0.60 mm longer before surgery, 1.00 mm longer after 1 month, and 0.50 mm longer at 6-month follow-up time (Table 4).

**Table 4:** Comparison between the two study groups in the internal and external length of the upper lip in both smile and rest positions at the evaluation periods

Variable	Study groups		CO_2_ laser group			Scalpel group			Mean difference	95 confidence interval of the difference		*p*-value
			Mean	*SD*	Median	Mean	*SD*	Median		Lower	Upper	
The internal length of the lip	Smiling position	Before surgery	9.70	3.06	9.5	10.50	2.59	11	− 0.80	− 3.46	1.86	0.341b
		1 month	6.20	1.75	6	7.20	1.87	7	− 1.00	− 2.70	0.70	0.233a
		3 months	6.80	1.75	7	7.80	1.62	8	− 1.00	− 2.58	0.58	0.201a
		6 months	7.10	2.77	7.5	7.70	1.89	7.5	− 0.60	− 2.83	1.63	0.578a
	Rest position	Before surgery	14.10	3.35	13	16.90	3.00	17	− 2.80	− 5.79	0.19	0.064a
		1 month	10.10	1.73	10	11.90	2.60	11.5	− 1.80	− 3.88	0.28	0.085a
		3 months	9.70	1.57	10	12.60	3.57	11.5	− 2.90	− 5.49	− 0.31	0.019b*
		6 months	10.40	2.67	10	12.30	3.97	11	− 1.90	− 5.08	1.28	0.276b
The external length of the lip	Smiling position	Before surgery	14.80	2.30	14.5	14.20	2.04	14.5	0.60	− 1.44	2.64	0.545a
		1 month	17.10	1.29	17	14.80	1.32	15	2.30	1.08	3.52	0.002b*
		3 months	16.80	1.75	17	15.80	1.14	15	1.00	− 0.39	2.39	0.161b
		6 months	16.40	2.76	17.5	15.90	1.60	15.5	0.50	− 1.62	2.62	0.337b
	Rest position	Before surgery	20.00	1.76	20	19.70	1.95	20	0.30	− 1.44	2.05	0.720b
		1 month	20.90	1.85	20	18.90	2.18	19.5	2.00	− 0.10	3.90	0.073b
		3 months	21.10	2.18	20	19.60	1.58	20	1.50	− 0.29	3.29	0.144b
		6 months	20.70	3.23	20.5	20.40	2.07	20.5	0.30	− 2.25	2.85	0.808a

^a^Independent samples *T*-test. ^b^Mann-Whitney test. Significant in *, 0.0

Regarding the internal upper lip length during smiling (the upper lip vestibule depth), the study found that no statistical difference was notable between the two groups at any evaluation point.

Zero complications were reported in either group. The average internal upper lip length when smiling in the surgical scalpel group was 0.80 mm longer before surgery, 1.00 mm longer at 1 and 3 months after surgery, and 0.60 mm longer at 6-month post-surgery. However, when analyzing the internal upper lip length at rest, the scalpel group elaborated a significantly deeper lip vestibule only at the 3-month follow-up, where it was 2.90 mm longer than the CO_2_ laser group (*p*-value < 0.05). Over evaluation periods, the difference was as follows: 2.80 mm longer before surgery, 1.80 mm deeper at 1-month post-surgery, and 1.90 mm deeper at 6-month follow-up point. These findings emphasized that the difference between the two groups was noticeable and statistically significant only at the 3-month follow-up evaluation time at rest position (Table 4).

## Discussion

Recently, the demand for achieving facial and dental esthetics has grown rapidly, and scientific literature is rich with traditional surgical techniques. Improving surgical techniques is crucial to better match patients’ requirements and satisfaction. However, numerous studies now evaluate dental lasers in soft-tissue surgery to accelerate healing and improve patient comfort. In general, laser use generally provides better coagulation, less edema, and inflammation by sealing lymphatic and blood vessels and causing less injury to myofibroblasts than scalpel incisions. All the benefits mentioned earlier play a vital role in leading to clinically measurable reduction in postoperative complications and enhancing patients acceptance [11, 21].

Postoperative pain is minimal in CO_2_ laser procedures; this is believed to be correlated to protein coagulum formation on the wound surface, which acts as a natural dressing and seals sensory nerve endings [22].

These advantages have practical and clinical significance, as the first days after oral surgeries are considered to impact patients’ quality of life. Therefore, decreasing postoperative complications is a critical key factor to patient comfort [23].

While the literature is full of studies regarding EGD treatment modalities, there is a lack in laser-assisted lip repositioning RCT studies. Reviewing literature shows some diode laser studies (mainly 940 nm), mostly case reports or series, demonstrating less invasive LRS than a conventional scalpel. These studies also emphasized diode laser’s role in offering esthetic results and lowering postoperative complications. They also reported reduced recurrence rates and improved comfort using various wavelengths for micro abrasion peeling [24–26]; this was different with the CO_2_ laser, where vaporization was used to cut the strip with no contact with tissues; this decreases the postoperative pain, minimizes exposure time and space, and consequently reduces the thermal effect. This results in vital, well-vascularized tissue, leading to accelerated healing at the surgical site. Another study utilized the Er, Cr, and YSGG laser (2780 nm) in LRS, with 1 W and 30 pulses per second settings; it showed reduced gingival display and improved patient satisfaction [27]. The CO_2_ laser’s advantage lies in its focused mode, providing a small spot size that enhances cutting accuracy, vaporization, tissue repair, and hemostasis. These factors allow the CO_2_ laser to reduce postoperative complications through precise cutting and avoiding insufficient over-or-under excisions [28]. On the other hand, in fiber-mode lasers such as diodes, cellular debris can stick to the hot fiber tip, absorbing energy and causing excess heat and collateral damage [11]; together with its 10,600-nm wavelength being highly absorbed by water-rich tissues [11], this thermal damage has been shown to be reduced in CO_2_ laser surgeries, making it suitable for precise oral [29]. In light of all the facts mentioned earlier, it was an opportunity to experimentally assess the role of the CO_2_ laser in LRS and offer evidence-based recommendations for its esthetic use. Additionally, it investigates the hypothesis that the CO₂ laser is a safe, effective alternative to the more affordable conventional scalpel. Overall, the CO_2_ laser provides a highly visualized surgical field when compared with the conventional scalpel, especially in extremely vascularized sites such as the upper lip. This is the first study to evaluate the CO₂ laser as an alternative to the scalpel in LRS. It is also among the few RCTs assessing upper-lip dimensional changes postoperatively, not just EGD correction.

This study demonstrated the CO₂ laser’s role in reducing postoperative pain. Hence, it may help make oral soft tissue surgery more conservative. Moreover, the CO₂ laser also reduces bacterial load and disinfects wounds through its thermal effect [30]. This may reduce the need for antibiotics after surgery and aligns with global concerns about antibiotic overuse. Although antibiotics were routinely prescribed postoperatively to minimize potential infections in several previous LRS studies [13, 31, 32], it might be considered now as unnecessary, as bacterial resistance is becoming a matter of growing concern. Additionally, other types of lasers have been recently utilized in performing laser-assisted LRS. For example, a diode laser (940 nm) was used to mark incision outlines in continuous-wave mode at 0.8 W, with the power increased to 1 W for tissue ablation. These parameters showed promising results in managing excessive gingival display [33]. Another case report demonstrated diode laser ablation at 1.2 W in continuous mode, achieving successful outcomes in terms of patient comfort and satisfaction [34]. However, it is important to note that studies on laser-assisted LRS remain limited in number and often involve small sample sizes, highlighting the need for further evidence to support the routine use of lasers in LRS.

The study suggests that CO₂ laser and scalpel offer comparable efficacy in LRS. The scalpel has been used for over 50 years to reduce gingival display. Our findings confirm that CO₂-assisted LRS reduces gingival display over time. The results showed no significant difference compared to traditional scalpel use. This represents a critical clinical significance, in which LRS performed utilizing CO_2_ laser may offer the same outcomes regarding managing excessive gingival display. Previous clinical and psychological studies have evaluated LRS impact on the social appearance anxiety scale (SAAS); the outcomes indicated a reduction in patients’ social anxiety after lip repositioning surgery [35].

Our findings align with the original LRS concept, removing a mucosal strip to reduce internal upper lip length, which is defined as the vertical measurement from the depth of the maxillary vestibule to the inferior border of the upper lip. Moreover, the study results found no significant change in total external upper lip length at rest, consistent with previous studies. This length extends from the base of the nose (subnasale) to the lower border of the upper lip. This finding, in its turn, represents an important clinical significance in reassuring patients’ concerns regarding the stability of their facial appearance after surgery. The documented changes in lip dimensional measurements provide assistance and guidance for both practitioners and patients in terms of treatment planning for LRS and patients counselling [14]; there are no significant differences between the two groups, except in the 1-month follow-up period of the smile position, and this may be due to the partial flap thickness without dissecting muscular tissue. Such significant difference at this follow-up point may be due to less tension and shrinkage in the early soft tissue wound healing.

Statistical analysis of the upper lip length indicated significant reduction in the vestibular depth through the follow-up period, similar to the results of [14]. No significant differences were found between groups, except in the 3-month follow-up in the at-rest position. This may be due to the controlled amount of removed tissues.

This study findings will open the door for future studies into the use of this laser as a more conservative alternative to the scalpel in LRS. Although LRS remains the gold standard for managing soft tissue-related EGD, proper case selection and diagnosis are key to achieving pleasing and stable outcomes [36]. However, several modifications have been introduced in the literature to reduce relapse and enhance stability, including myotomy or using spacers [37].

### Study limitations

Study limitations include the relatively short follow-up period and the small sample size. Despite being the gold standard for soft tissue EGD, long-term LRS results remain prone to relapse. A further limitation is the patient compliance to postoperative instructions, especially regarding muscle memory and avoiding lip movement in the 2 weeks following surgery, which may affect the results stability.

Robust outcome of this study may be affected by the study’s small sample size (20 patients) and single-center setting at the Faculty of Dentistry and clinical evaluations being performed by a single examiner. Therefore, future studies should consider involving multiple calibrated examiners for postoperative assessments. All these factors may limit its application to a broader population. While the controlled academic environment ensured standardized procedures, real-world variations in practitioner expertise, equipment availability, and patient factors could influence the findings. Moreover, the inclusion criteria may not fully represent patients with diverse excessive gingival display underlying factors or patients’ medical conditions. Although randomization in this study was performed using sealed, opaque envelopes to ensure allocation concealment, this traditional method may have limitations regarding potential selection bias. Future studies could benefit from employing computer-generated randomization software with allocation concealment to further improve the robustness. Despite its limitations, this study offers valuable insights into using the CO_2_ laser versus the conventional scalpel in performing LRS for gummy smile correction, though longer follow-up and larger multicenter trials are needed to confirm long-term effectiveness and broader applicability.

## Conclusion

Within the limitations of this current study, it was concluded that this randomized controlled clinical trial has sought statistical significance in comparing the surgical scalpel and carbon dioxide laser in performing a lip repositioning technique to reduce excessive gingival display. The results obtained together with statistical analysis would suggest that the CO_2_ laser is an effective, safe tool in performing LRS to manage EGD with soft-tissue origins. A novel advantage of utilizing this laser has been demonstrated to render LRS as more conservative by decreasing pain levels and performing the whole surgery with no noticeable bleeding and to optimize visual control of the procedure. The encouraging outcome of this study offers scope to expand the group cohort numbers and longer postoperative periods of assessment in order to further endorse the evidence base of laser-assisted oral soft tissue surgical management and predictability. There is a need to exercise due care when employing surgical laser technology, and the authors recommend using the CO_2_ laser only after adequate training by dental laser experts and awareness of laser-tissue interaction and safety. Though our study resulted in comparable effectiveness between laser and scalpel in LRS standard technique, we hope our findings will open the door for future research in which CO_2_ laser may be explored in LRS-modified techniques.

## Data Availability

Data is provided within the manuscript or supplementary information files.

## Abbreviations

CO₂: Carbon dioxide
LRS: Lip repositioning surgery
VAS: Visual analog scale
EGD: Excessive gingival display
